# On-demand self-care products: a systematic scoping review of user perspectives

**DOI:** 10.1080/26410397.2026.2660555

**Published:** 2026-05-18

**Authors:** Stephen Bell, Susannah Gibbs, Claire W. Rothschild, Heather Ingold, Lemessa Oljira, Irene Akhigbe, Dinesh Kumar, Marion Subah, Leora Nicole Pillay, Kanwal Qayyum, Eden Demise, Sarah Onyango, Abigail Winskell, Kristen Little

**Affiliations:** a Senior Principal Research Fellow; Co-Head, Global Adolescent Health; Head, International Development, Burnet Institute, Melbourne, Australia; b Senior Technical Writer, Public Health Research, Strategy & Insights Department, Population Services International, Washington, DC, USA; c Senior Research Advisor II, Sexual and Reproductive Health Department, PSI, Washington, DC, USA; d Programme Manager, Global HIV, Hepatitis, and STI Programmes, World Health Organisation, Geneva, Switzerland; e Associate Professor, Haramaya University, Ethiopia; f Consultant, Department of Paediatrics, Ola During Children’s Hospital, and Ministry of Health and Sanitation, Freetown, Sierra Leone; g Professor of Community Medicine, Dr. Rajendra Prasad Government Medical College, Chandigarh, India; h Country Director, Last Mile Health, Monrovia, Liberia; i Lead, HIV Prevention Advocacy, Frontline AIDS, Manchester, UK; j Senior Research Manager, Jhpiego Pakistan, Islamabad, Pakistan; k Research Advisor I, Strategy & Insights Department, Population Services International, Washington, DC, USA; l Senior Technical Advisor II, Self-Care, Health Systems Accelerator, Population Services International, Washington, DC, USA; m Project Director, Multi-Country II, Sexual and Reproductive Health Department, Population Services International, Washington, DC, USA; n Senior Research Advisor II, Strategy & Insights Department, Population Services International, PSI, 1120 19th Street, NW Suite 600, 20036 Washington DC, USA. *Correspondence*: klittle@psi.org

**Keywords:** on-demand, self-care, sexual and reproductive health, contraception, family planning, HIV prevention, PrEP, acceptability, community perspectives, consumer insight

## Abstract

On-demand sexual and reproductive health (SRH) products are used or consumed on an as-needed basis, around specific and immediate sexual events, to reduce the risk of unwanted health outcomes. To date, understandings of how on-demand SRH products contribute to self-care are relatively under-explored. Our systematic scoping review of available published research explored acceptability of SRH on-demand self-care from the perspectives of users across diverse regions of the world. Studies from 20 countries across four WHO regions indicated strong acceptability of on-demand self-care SRH products due to compatibility with sex lives; ease of use; fewer side effects; accessibility of products; compatibility with daily life; and partner dynamics related to product use. Interest in and experiences with on-demand products varies for people in different phases of their life course. On-demand SRH products enable self-care through enhanced choice and agency; people-centred products and SRH service delivery; and more pleasurable, safer approaches to SRH. They support users to have satisfying sex lives, plan the families they want and stay healthy. The six emergent domains we identify can be used to position on-demand products into the market; engage and communicate with end-users in ways that enhance their use of safe, effective products most aligned to their needs and preferences across the life course; advocate for changes in policy and regulations that are more supportive of on-demand SRH products; and prioritise future investment in the on-demand product category.

## Plain language summary

People can use on-demand sexual and reproductive health (SRH) self-care products just before, during, or after sex to reduce their risk of unwanted health outcomes like pregnancy or HIV. On-demand products can be used only if and when care is actually needed, putting SRH care more in users' control. To date, there has not been much research on how these products contribute to self-care. We carried out a systematic scoping review of the published research on how users in different regions of the world perceive SRH on-demand self-care products. We identified studies from 20 countries across four WHO regions, that show that on-demand self-care SRH products are very acceptable. Across product categories, on-demand self-care interventions were compatible with users' daily lives. They were seen as easy to access and to use. These products also aligned well with users' sex lives. They didn't negatively affect relationships with partners, and enabled users to plan the families and relationships they wanted. Interest in and experiences with on-demand products varies at different stages of people's lives and reproductive and sexual health journeys. The literature suggests that on-demand SRH products enable self-care by giving people greater choice and agency, and by promoting more pleasurable, safer approaches to SRH. This form of care allows people to choose whether and how they want to engage with formal health services. For many people, access to and use of on-demand SRH self-care products put the pleasure back into SRH.

## Résumé

Les produits de santé sexuelle et reproductive (SSR) à la demande sont utilisés ou consommés selon les besoins, lors d’événements sexuels spécifiques et immédiats, afin de réduire les conséquences indésirables pour la santé. À ce jour, la manière dont ces produits contribuent à l’auto-prise en charge est relativement peu étudiée. Notre étude systématique de la portée des recherches publiées a examiné l’acceptabilité de l’auto-prise en charge de la SSR à la demande du point de vue des utilisateurs dans différentes régions du monde. Des études menées dans 20 pays répartis dans quatre régions de l’OMS ont révélé une forte acceptabilité de ces produits, notamment du fait de leur compatibilité avec la vie sexuelle ; leur facilité d’emploi ; la réduction des effets secondaires ; leur accessibilité ; leur intégration dans la vie quotidienne; et la dynamique de la relation de couple liée à leur utilisation. L’intérêt pour ces produits et l’expérience des personnes varient selon les différentes étapes de leur vie. Les produits de SSR à la demande permettent une auto-prise en charge grâce à un choix élargi et de plus grandes possibilités d’action; des produits et des services de SSR centrés sur les personnes; ainsi que des approches plus agréables et plus sûres en matière de SSR. Ils permettent aux utilisateurs d’avoir une vie sexuelle épanouie, de planifier le nombre d’enfants qu’ils souhaitent et de rester en bonne santé. Les six domaines émergents que nous avons identifiés peuvent être utilisés pour positionner les produits à la demande sur le marché; interagir et communiquer avec les utilisateurs finals de manière à améliorer leur utilisation de produits sûrs et efficaces, mieux adaptés à leurs besoins et préférences tout au long de leur vie; plaider en faveur de changements de politiques et de réglementations plus favorables aux produits de SSR à la demande; et prioriser les investissements futurs dans la catégorie des produits à la demande.

## Resumen

Los productos de salud sexual y reproductiva (SSR) bajo demanda son utilizados o consumidos según sea necesario, en torno a sucesos sexuales específicos e inmediatos, para reducir el riesgo de resultados de salud no deseados. Hasta la fecha, se ha examinado con relativa poca frecuencia la comprensión de cómo los productos de SSR bajo demanda contribuyen al autocuidado. Nuestra revisión exploratoria sistemática de investigaciones publicadas disponibles exploró la aceptabilidad del autocuidado de SSR bajo demanda desde las perspectivas de usuarias en diversas regiones del mundo. Estudios realizados en 20 países de cuatro regiones de la OMS indicaron una gran aceptabilidad de los productos de autocuidado de SSR bajo demanda debido a su compatibilidad con la vida sexual de las usuarias; facilidad de uso; menos efectos secundarios; accesibilidad de los productos; compatibilidad con la vida diaria; y las dinámicas de pareja relacionadas con el uso de los productos. El interés en los productos bajo demanda y las experiencias con ellos varían según cada persona en diferentes fases de su vida. Los productos de SSR bajo demanda facilitan el autocuidado mediante mejores opciones y agencia; productos y prestación de servicios de SSR centrados en la persona; y enfoques de SSR más placenteros y seguros. Apoyan a cada usuaria en tener una vida sexual satisfactoria, planificar la familia que desea y mantenerse saludable. Los seis ámbitos emergentes que identificamos pueden utilizarse para posicionar los productos bajo demanda en el mercado; interactuar y comunicarnos con las usuarias finales en maneras que mejoren su uso de los productos seguros y eficaces más alineados con sus necesidades y preferencias a lo largo de su vida; abogar por cambios en políticas y normativas que apoyen más el uso de productos de SSR bajo demanda; y priorizar la inversión futura en la categoría de productos bajo demanda.

## Introduction

Though practised socially and culturally around the world for millennia, the World Health Organization (WHO) formalised the idea of sexual and reproductive (SRH) self-care in 2019 (updated in 2022).^1,2^ This recognised the diverse ways in which people practise self-management, self-testing and self-awareness for improved health and wellbeing. WHO defines self-care interventions for SRH as “tools” that include “evidence-based, high-quality drugs, devices, diagnostics and/or digital interventions that can be provided fully or partially outside formal health services”.^2^ The emergence of new health self-care interventions for SRH supports the transition of traditional models of facility or provider-based care into community settings, households and the hands of end-users. Such interventions support self-care, which has been defined broadly as “the ability of individuals, families and communities to promote health, prevent disease, maintain health and cope with illness and disability with or without the support of a health worker”.^2^ Self-care interventions have been lauded as a strategy to overcome the challenges of achieving and sustaining universal health coverage by enhancing access, choice and agency, and extending health system reach to underserved populations through innovation beyond health facilities within communities and homes.^3–5^

Self-care interventions can support people-centred approaches to SRH.^5^ People-centred approaches prioritise individuals’ needs, preferences, and values while empowering them as active participants in their care, and are fundamental to achieving sustainable universal health coverage by ensuring health systems are designed and delivered for and with people.^6^ In relation to SRH, this is achieved through key characteristics of self-care, which include, but are not limited to: improved flexibility, convenience, opportunity and choice in the way people access and seek SRH care and support; attentiveness to need and diversity of experience by age, gender, sexuality, race and culture; enabling agency to help marginalised peoples navigate socio-cultural contexts of constraint, stigma and discrimination, or self-manage SRH care with or without the support of a healthcare worker; enabling interaction with health systems and services as needed; enhanced availability, accessibility and affordability of SRH care; greater self-efficacy, autonomy and engagement in health for self-carers and caregivers; and improved health, human rights and social outcomes.^2,3,5,7^

Our interest in this paper is in self-care interventions oriented around a product category known as “on-demand”. With regard to SRH, on-demand products are those used or consumed on an as-needed basis, around specific and immediate sexual events, to reduce the risk of unwanted health outcomes (i.e. pregnancy, HIV infection). Examples of on-demand SRH products in the WHO’s current guideline^2^ include male and female condoms, emergency contraceptive pills (over-the-counter, without a prescription), event-driven indications for oral pre-exposure prophylaxis (ED-PrEP), and use of lubricants. We are interested in on-demand SRH products as these extend the concept of self-care by giving users tools that they can use if and when care is actually needed, thereby pushing SRH care closer to end-users themselves.

To date, understandings of how on-demand SRH products as a new category contribute to self-care are relatively under-explored, and restricted to product-specific studies.^8^ The objective of this paper is to examine the acceptability of SRH on-demand self-care from the perspectives of users across diverse places, for different people with different needs, and across the life course. To do this, we used a systematic scoping review methodology to analyse published literature reporting end-user acceptability of four illustrative SRH products that can be used on-demand: event-driven pre-exposure prophylaxis (ED-PrEP); on-demand contraceptive pills (ODCPs), which are also referred to as pericoital oral contraceptive pills in other literature; vaginal gels and films; and single-size diaphragms and cervical caps.

## Systematic scoping review methodology

### Objective and research questions

Our objective was to undertake a comprehensive review of available published research to explore the acceptability of SRH on-demand self-care from the perspectives of users across diverse regions of the world. In the context of people’s sexual lives, we define acceptability as whether people are willing to use a product, and whether products align with their lives, values, sexual experiences and preferences.^9^ Using an inductive analysis approach, this scoping review addresses two main research questions: (1) What are the factors driving acceptability and use of on-demand self-care products? and (2) How does use of on-demand SRH products enable self-care?

### Review approach

In 2005, Arksey and O’Malley defined a scoping review as a structured, transparent and rigorous method to synthesise and analyse published literature and identify knowledge and research gaps.^10^ This method typically addressed broad research questions to present an overview and organisation of existing knowledge, rather than a narrow synthesis of a predefined research question, and comprised several stages: identifying a research question or topic; identifying relevant studies; study selection; synthesising and interpreting data; and summarising and reporting on the results.^10^ In 2015, Peters et al^11^ published a methodology and guidance for the conduct of *systematic* scoping reviews, to improve the utility and robustness of the results of scoping reviews; we have followed this process in the development of this paper. This review was guided by a review protocol,^12^ and prepared in accordance with the guidance laid out in the PRISMA Extension for Scoping Reviews (see Supplemental Material).^13^

Our review focused on end-user perspectives of four on-demand product types across the SRH space: ED-PrEP, to prevent HIV acquisition; ODCPs to prevent pregnancy; single-size diaphragms (e.g. SILCS, Caya) and cervical caps to prevent pregnancy; and multi-purpose vaginal gels and films to protect against pregnancy, HIV or other STIs. Product characteristics are detailed in Table 1. These four product categories were chosen to illustrate a range of on-demand SRH self-care products, with heterogeneity in terms of time on market, published evidence base, target population and type of protection afforded, and which enabled discrete or covert use to maximise user agency. Products were selected in collaboration with members of an expert working group assembled by the Self-Care Trailblazers Group’s Evidence and Learning Working Group (SCTG ELWG).^14^ Male condoms were excluded because the literature was considered too vast to review in this context. Literature on postcoital use of emergency contraceptive pills was excluded to avoid repetition of analyses presented in a separate review.^8^ WHO recommendations about ED-PrEP changed in July 2024,^15^ moving to recommending two dosing regimens for TDF-based oral PrEP depending on the person’s characteristics, circumstances and the route of exposure. However, this review of published literature on ED-PrEP supported our analysis and exploration of on-demand SRH products as a new category.

**Table 1.** Summary of selected on-demand SRH product characteristics

**Product type**		Product description
1.	Event-driven (ED) PrEP	Sexual activity-based, indicated for cisgender men who have sex with men and transgender women (not taking gender-affirming hormones); Truvada pill (tenofir disoprxil fumarate/emtricitabine); two pills taken between 2 and 24 hours preceding sexual intercourse, followed by one pill 24 hours and another pill 48 hours after the first drug intake
2.	On-demand contraceptive pill (ODCP)	Sexual activity based (i.e. event-driven); Levonorgestrel 1.5 mg tablets*; only one pill taken within 24 hours before or after sexual intercourse, and no more than six times per month.
3.	Vaginal gels and films	Spermicide products that, once inserted into the vagina, kill sperm; used prior to sexual intercourse
4.	Single-size diaphragms	Includes products known as SILCS or Caya; female-controlled barrier method; silicone diaphragm, manufactured to have a more anatomical shape than traditional dome diaphragms, with an oval contoured shape, flexible rim, grip dimples and a removal dome; placed in the vagina so that it covers the cervix (entrance to the uterus), tucks in behind the pubic bone and is held in position by the pelvic muscles; estimated to fit approximately 80% of women
	Cervical cap	Includes a product known as Femcap; female-initiated, coitally dependent, nonhormonal method of contraception; a small, flexible silicone device inserted onto the cervix that adheres via gentle suction and can be worn for 48 hours continuously to provide contraceptive protection; one initial application of spermicide inside the device is required prior to placement; used at home after guidance from health provider

*We note that while we include ODCPs using the LNG 1.5 product, there are other formulations being explored, including LNG in combination with other medications, or entirely different drugs.

### Identification of studies

Four databases were searched on 8–9 December 2024 to identify papers relevant to the research questions: PubMed; Web of Science; Global Index Medicus; Scopus. Search terms used for four different searches (i.e. one per product category) are detailed in Table 2. We also searched Google Scholar using similar terms and reviewed the first ten pages of results and reviewed the citations from relevant reviews uncovered during the literature search. The results were limited to human studies reporting primary data or original analysis of secondary data published in peer reviewed journals between 2014 and 2024. We included all qualitative, quantitative and mixed methods studies that reported preference, acceptability and usability data from the perspectives of end-users. Inclusion criteria were not restricted by geographic location or publication language. During review, papers were excluded if the research was not peer reviewed; did not contain primary data/original analysis of secondary data; or did not include the use of a selected product on an on-demand basis. Unpublished grey literature, conference abstracts/ reports, letters, media articles, cost-effectiveness and bench science studies were also excluded.

**Table 2.** Search terms.

Product type	Search terms
ED-PrEP	(PrEP OR “pre-exposure prophylaxis”) AND (intermittent OR non-daily OR nondaily OR coitally-related OR “fixed dose” OR post-coital OR “on demand” OR event-based OR event-driven OR time-driven OR weekly-based OR routine OR periodic)
ODCPs	(“on demand” OR on-demand OR pericoital OR peri-coital OR precoital OR pre-coital OR repeat* OR routine) AND (contraception OR contraceptive OR “family planning” OR “emergency contraception” OR “emergency contraceptive” OR levonorgestrel OR lng OR postinor* OR “morning after pill” OR “morning after pills” OR “plan b”)
Single-size diaphragm and cervical cap	((silcs OR caya OR single-size) AND diaphragm) OR “cervical cap”)
Vaginal gels and films	(gel OR film OR “vaginal cream*” OR “vaginal foam*” OR “vaginal jell*”) AND (contraception OR contraceptive OR “family planning”)

Per product category, records were deduplicated across databases, and title/abstract dual review was conducted in pairs by members of the research team (KL, SB, SG). Reviewers discussed and resolved any discordances and recorded an exclusion reason for each record. We then obtained the full text of all articles identified as potentially relevant during the title/abstract review and proceeded to a combined round of full text review for inclusion and data extraction (KL, SB).

### Data extraction and synthesis

The final selections of papers identified for inclusion in each of the four categories were pooled together; uploaded into the data management tool (NVivo X9) in preparation for synthesis and thematic analysis; and reviewed using a data extraction tool designed by the authors for this scoping review. Two types of information were collected from each paper guided by data extraction fields outlined in Table 3.

**Table 3.** Data extraction fields

Journal information & study overview	On-demand self-care product information
Publication citation Study setting Population characteristics Dates of data collection Sample size Analysis technique Theoretical framework Ethical considerations	Definitions of ‘self-care’ and ‘on-demand’ Descriptions of one or more of the four eligible products, and any information regarding appropriate use Factors that enhance acceptability/use Factors that inhibit acceptability/use Other relevant info

Primary data reported in each manuscript were analysed using inductive thematic techniques following Strauss and Corbin’s^16^ system of “open” and “axial” coding. First, data familiarisation occurred through reading and re-reading the manuscripts, and open codes were attributed to data. During this process, “theoretical memos”^16^ were prepared as analytical reminders for generating ideas and making links between different codes. Second, axial coding techniques were used to link or organise open codes into emergent themes and sub-themes. During this process, we collated evidence against a set of emerging “domains” that were perceived to influence the acceptability of on-demand self-care products and help to understand how on-demand products enable SRH self-care. The following findings section reports on six emergent domains that came out of inductive coding.

## Results

**Figure 1.**
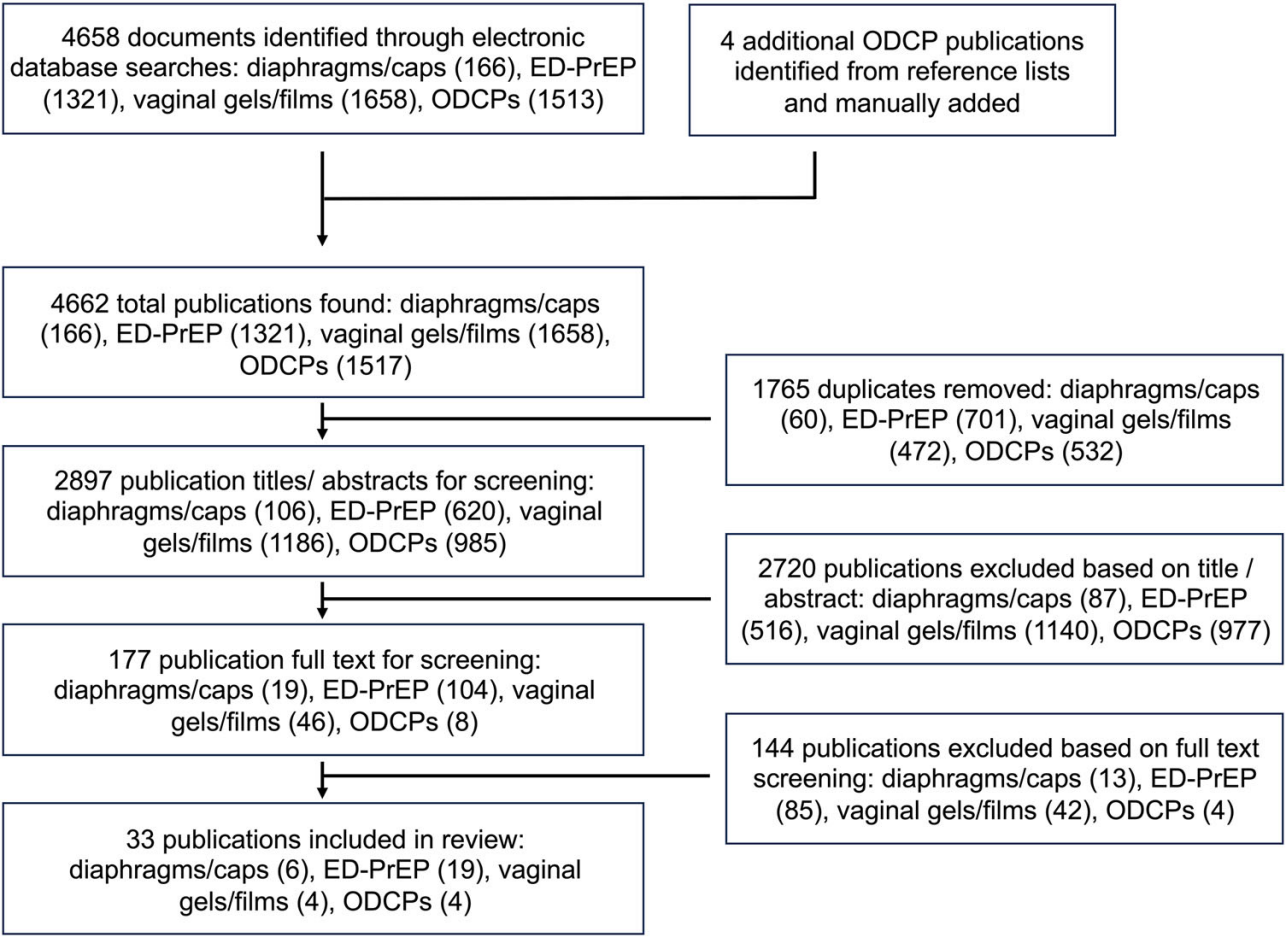
Flow chart showing the selection process

A total of 2897 unique references were identified. After screening, 33 papers met the inclusion criteria for the scoping review (see Figure 1).

The characteristics of the final 33 papers are summarised in Table 4. With regard to the type of on-demand product, 19 papers reported on ED-PrEP, and were published between 2018 and 2024^23–41^; six papers reported on diaphragms/cervical caps, and were published between 2014 and 2024^17–22^; four papers reported on vaginal gels and films, and were published between 2020 and 2022^42–45^; and four papers reported on ODCPs, and were published between 2014 and 2023^46–49^.

**Table 4.** Summary of paper characteristics

Citation	Study design	Study population and sample size	Location of study	Domain
**Diaphragms/cervical caps**				
Kyamwanga *et al* 2014^17^	SILCS diaphragm; qualitative: focus groups	Women of reproductive age (*n* = 201), and their male partners (*n* = 77)	Uganda	1,2,3,4,5,6
Gollub *et al* 2015^18^	FemCap cervical cap; Mixed methods: survey, focus groups	Haitian/Haitian-American women, 19–45 years (*n* = 20)	USA	2,3,4,6
Gollub *et al* 2016^19^	FemCap cervical cap; qualitative: focus groups	Haitian/Haitian-American women, 19–45 years (*n* = 20)	USA	1,2,3,4,5,6
Bekinska *et al* 2018^20^	SILCS diaphragm; quantitative: survey (within non-blinded randomized crossover study)	Women, 18–45 years (*n* = 115)	South Africa	1,4,6
Jackson *et al* 2022^21^	Caya diaphragm; mixed methods: survey; in-depth interviews	Women, aged 18–49 years (*n* = 175); Men (*n* = 25)	Niger	1,2,3,4,5,6
Zinsou *et al* 2024^22^	Caya diaphragm; mixed methods: survey; in-depth interviews	Women, aged 18–49 years (*n* = 225); Men (*n* = 15); Providers (*n* = 15)	Benin	1,3,6
**ED-PrEP**				
Dubov *et al* 2018^23^	Quantitative: survey (choice-based conjoint)	Men who have sex with men (*n* = 1184)	Ukraine	4,5
Patel *et al* 2018^24^	Mixed methods: survey, in-depth interviews	Young adult African American Men who have sex with men, 18–35 years (*n* = 26)	USA	1,2
Reyniers *et al* 2018^25^	Mixed methods: survey, in-depth interviews (within open-label prospective cohort study)	Men who have sex with men (*n* = 200)	Belgium	2,3
Zimmermann *et al* 2019^26^	Qualitative: in-depth interviews (within prospective, longitudinal, open-label demonstration project)	Men who have sex with men (*n* = 374) and transgender persons (*n* = 2)	The Netherlands	1,2,3,4
Puppo *et al* 2020^27^	Qualitative: in-depth interviews, focus groups (within randomized clinical trial ANRS-IPERGAY)	Men and transgender women who have sex with men (*n* = 83)	France	1,2,
Camp & Saberi 2021^28^	Quantitative: survey	Men who have sex with men (*n* = 140)	USA	1,2,3,4
Dietrich *et al* 2021^29^	Qualitative: in-depth interviews, focus groups	Young men (*n* = 38) and young women (*n* = 36), 13–24 years (*n* = 74)	South Africa	1,2,4
Kawuma *et al* 2022^30^	Qualitative: in-depth interviews, focus groups	Young men (*n* = 45) and young women (*n* = 57), 14–19 years	Uganda	1,2,5
Philpot *et al* 2022^31^	Qualitative: in-depth interviews	Gay, bisexual and queer men (*n* = 40)	Australia	1,2,3,4
Biello *et al* 2023^32^	Quantitative: survey	Young men who have sex with men, 15–24 years (*n* = 737)	USA	1,3
Dietrich *et al* 2023^33^	Qualitative: in-depth interviews, focus groups	Young men (*n* = 93) and women (*n* = 96), 13–24 years	South Africa; Uganda; Zimbabwe	1,2,3,5
Dubov *et al* 2023^34^	Quantitative: survey (discrete choice)	Men who have sex with men (*n* = 718)	Malaysia	4,5
Horvath *et al* 2023^35^	Quantitative: survey (within longitudinal intervention study)	Young adult gay, bisexual and other men who have sex with men, 18–29 years (*n* = 80)	USA	1,2,3,5
Kakande *et al* 2023^36^	Mixed methods: survey, in-depth interviews, focus groups	Young men, 13–24 years (*n* = 647)	South Africa; Uganda; Zimbabwe	1,2,5
Smith *et al* 2023^37^	Qualitative: in-depth interviews	PrEP users, aged 18+ (*n* = 40)	Australia	1,2,3,4,6
Nguyen *et al* 2024^38^	Quantitative: survey (within prospective cohort study)	Men who have sex with men (*n* = 926)	Viet Nam	1,2,4
Oliveri *et al* 2024^39^	Qualitative: in-depth interviews, focus groups	HIV key populations (*n* = 63)	Cambodia	1,2,3,4,5
Daroya *et al* 2024^40^	Qualitative: longitudinal in-depth interviews	Gay, bisexual and queer men (*n* = 38)	Canada	2,3
Deus *et al* 2024^41^	Qualitative: in-depth interviews	Young men who have sex with men, *Travestis* and transgender women, aged 15–19 years (*n* = 50)	Brazil	1,2
**Vaginal gels and films**				
Thomas *et al* 2020^42^	Gel; quantitative: survey (within single-arm, open-label, phase 3 clinical trial study)	Women, 18–35 years (*n* = 1384)	USA	3
Weinrib *et al* 2020^43^	Gel; mixed methods: survey, focus groups (within two-stage randomized cross-over acceptability study)	Young women, 18–30 years (*n* = 190)	South Africa; Zimbabwe	2,6
Minnis *et al* 2022^44^	Film; quantitative: survey (discrete choice)	Adult couples, including women, 18–40 years, and men, 18+ (*n* = 400 couples)	Uganda; Zimbabwe	3,5,6
Thomas *et al* 2022^45^	Gel; quantitative: survey (within single-arm, open-label, phase 3 clinical trial study)	Women, 18–35 years (*n* = 1330)	USA	6
**Pericoital or postcoital ODCPs**				
Chin-Quee *et al* 2014^46^	Peri-coital ODCP; quantitative: survey	Women, 18–49 years (*n* = 6162)	Kenya; Nigeria	1,3,5
Kalamar *et al* 2021^47^	Post-coital ODCP (i.e. emergency contraceptive pill); qualitative: in-depth interviews, focus groups	Women, 18–34 years (*n* = 299); men, 18–30 years (*n* = 75)	Ghana; Zambia	1,2,4,5
McCann *et al* 2023^48^	Peri-coital ODCP; quantitative: surveys (within 12-month prospective, single-arm, interventional study)	Women, 18–49 years (*n* = 873)	Ghana	1,2,3,4
Odwe *et al* 2023^49^	Peri-coital ODCP; quantitative: surveys (within 12-month prospective, single-arm, open-label interventional study)	Women, 18–49 years (*n* = 768)	Kenya	1,2,3,4

Methodologically, these papers used different study designs, reporting on research produced from qualitative (*n* = 12),^17,19,26,29–31,33,37,39–41,47^ quantitative (*n* = 14),^20,23,27,28,32,34,35,38,42,44–46,48,49^ and mixed methods (*n* = 7)^18,21,22,24,25,36,43^ studies.

**Figure 2.**
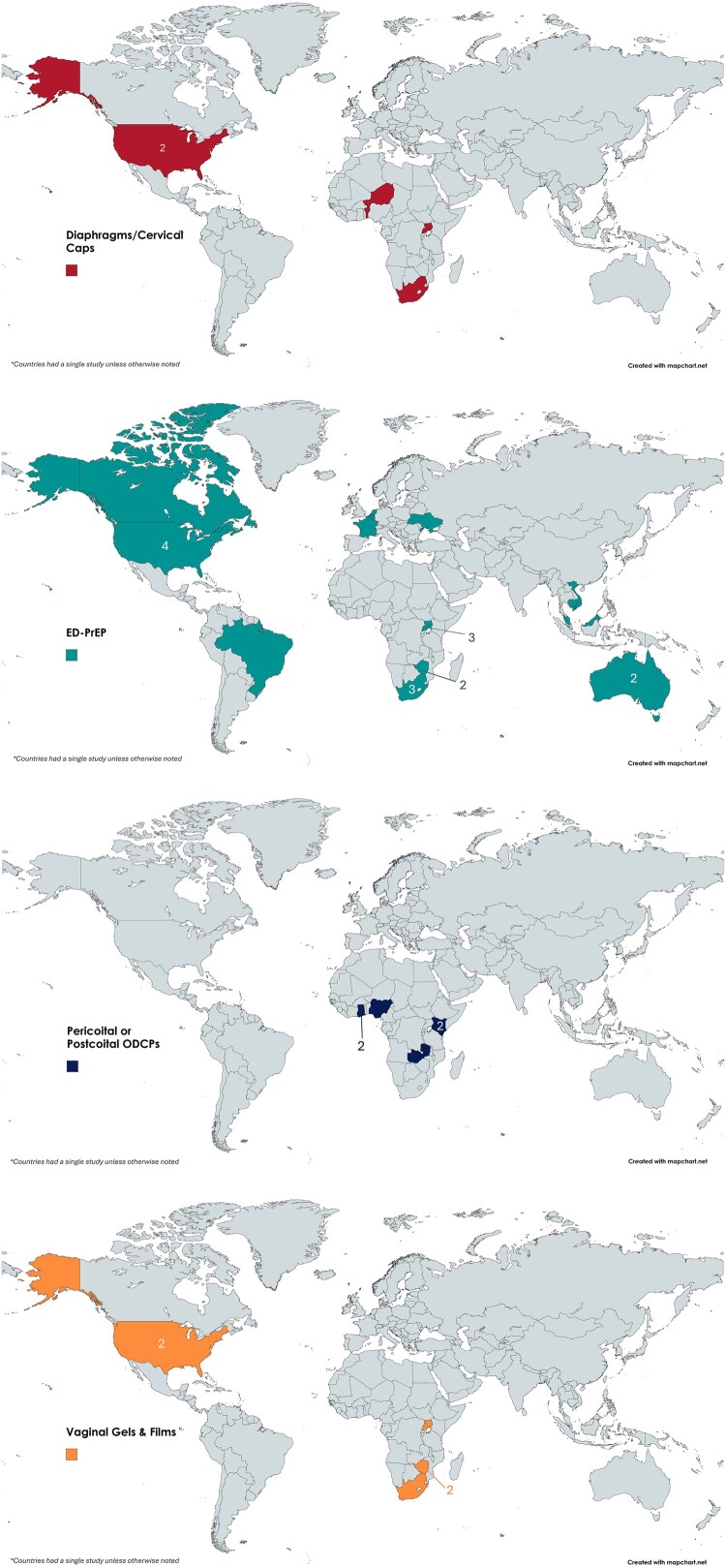
Countries where selected research was conducted across four on-demand SRH self-care products

Papers reported on research conducted in 20 countries across four WHO regions (see Figure 2): nine countries in Africa, including Benin,^22^ Ghana,^47,48^ Kenya,^46,49^ Niger,^21^ Nigeria,^46^ South Africa,^20,29,33,36,43^ Uganda,^17,30,33,36^ Zambia,^47^ and Zimbabwe;^30,33,36,43^ four countries in Western Pacific, in Australia,^31,37^ Cambodia,^39^ Malaysia^34^ and Vietnam;^38^ four countries in Europe, in Belgium,^25^ France,^27^ the Netherlands,^26^ and Ukraine;^23^ three countries in the Americas, in Brazil,^41^ Canada,^40^ and the US.^18,19,24,28,32,35,42,45^ There was variation in where research about each type of product was reported. For example, research about ED-PrEP was reported in countries in Europe, Africa, the Americas and Asia; research about diaphragms/cervical caps and vaginal gels was reported in countries in Africa and the Americas; while research about ODCPs was reported in countries in Africa. There were no papers reporting on research from South East Asia or Eastern Mediterranean Regions.

We identified six emergent domains that influence acceptability of on-demand self-care products and help to understand how on-demand products enable SRH self-care. In total, 25 papers reported on domain 1, ease of use;^17–22,24,26–33,35–39,41,46–49^ 23 papers reported on domain 2, compatibility with sex lives;^17,19,21,24–31,33,35–41,43,47–49^ 20 papers reported on domain 3, side effects;^17–19,21,22,25,26,28,31–33,35,37,39,40,42,44,46,48,49^ 17 papers reported on domain 4, accessibility of products;^17–21,23,26,28,29,31,34,37–39,47–49^ 13 papers reported on domain 5, compatibility with daily life;^17,19,21,23,30,33–36,39,44,46,47^ 10 papers reported on domain 6, partner dynamics with product use.^17–22,37,43–45^ See also Table 5.

**Table 5.** Mapping on-demand products against domains

Domain	ED-PrEP	ODCP	Diaphragms	Gels/films	Total studies
1. Ease of use	15 studies^24,26–33,35–39,41^	4 studies^46–49^	6 studies^17–21,22^	–	25
2. Compatibility with sex lives	16 studies^24–31,33,35–41^	3 studies^47–49^	3 studies^17,19,21^	1 study^43^	23
3. Side effects	10 studies^25,26,28,31–33,35,37,39,40^	3 studies^46,48,49^	5 studies^17–19,21,22^	2 studies^42,44^	20
4. Accessibility of products	9 studies^23,26,28,29,31,34,37–39^	3 studies^47–49^	5 studies^17–21^	–	17
5. Compatibility with daily life	7 studies^23,30,33–36,39^	2 studies^46,47^	3 studies^17,19,21^	1 study (42)	13
6. Partner dynamics with product use	1 study^37^	–	6 studies^17–22^	3 study^43–45^	10

### Examining acceptability of on-demand self-care products

#### Domain 1 – ease of use

Ease of use and convenience of products were noted in 25 studies, relating to diaphragms/caps,^17–22^ ED-PrEP^24,26–33,35–39,41^ and ODCPs.^46–49^ Study participants often discussed these in comparison to other available products. Characteristics that enhanced acceptability of diaphragms/ caps included product durability and reusability,^18,19,21^ flexibility of when the product can be used,^22^ ability to self-use the product,^22^ and the convenience of always having the product at home and ready to use, unlike condoms which had to be restocked.^21^ In a Caya study in Niger,^21^ 115/150 survey respondents (77%) reported continued use of Caya. Women described the method as “easy” to use in contrast with oral contraceptive pills (OCPs), which require daily action. One woman said, “*people won’t forget [Caya], unlike [OCPs] that we can forget to take*”.

Concerns about forgetting to take daily PrEP medication were reported among people who decided to use ED-PrEP instead to prevent HIV infection.^26,29,30,32,33,36^ Among young men who have sex with men (MSM) in a US study, the highest endorsed reasons for ranking ED-PrEP instead of other HIV prevention products included ease of use, ability to stop taking it, no impact on daily routines, and perceived protection against HIV.^32^ Young people in studies in South Africa, Zimbabwe and Uganda^33,36^ explained that use of ED-PrEP rather than daily PrEP avoided the issues of pill fatigue and forgetfulness. A young woman (20 years) in South Africa said, “*I feel like using [PrEP] only when you need it, because taking a pill is hard when you don’t feel, you are not sick, right? … Taking a pill is hard and it is forgettable”.*^33^

##### Learning to use a new product

Assistance with practical knowledge of how to use new products was perceived as important in seven papers.^18–21,31,48,49^ In research about diaphragms/caps, information sources included community health workers,^21^ facility-based providers,^20,21^ counsellors,^18,19^ and friends, relatives and neighbours.^21^ Demonstrations and support to learn how to fit the diaphragm/cap were typically provided by a healthcare provider.^18–21^ These studies illustrate the critical role providers continue to play in the uptake, correct and continued use of self-care interventions, even for on-demand products. Women involved in research about the SILCS diaphragm reported this type of support as essential when learning how to use a new product,^18,20^ driving feelings of self-efficacy for using the device at home on their own.^19^ In two ODCP trials, women received information from pharmacists, either face to face in Ghana^48^ or via a call centre in Kenya,^49^ who were trained in the provision of LNG 1.5 mg for pericoital use and provided use instructions to participants (e.g. maximum use per month guidelines, side effect reporting, and general use guidance) as they were dispensed. Both strategies were deemed acceptable and feasible by study participants.

##### On-demand products also require work and effort

Research indicates that using on-demand products is not necessarily always easy or convenient, may not be desirable for everyone, and may not be appropriate for people at all stages of their lives. Research on diaphragms/caps^17–21^ reveals some consumers work to understand how to use these products effectively. In a qualitative study with 20 Haitian-American women in the US,^18,19^ initially most women found it hard and uncomfortable to remove the cervical cap due to the need to find the correct place to put pressure with the finger to break the cap’s suction seal. Difficulties with removal reduced over time as women gained experience.^18^

While many study participants reported liking the lower pill burden of ED-PrEP, the complexity of the dosing regimen dissuaded some participants from using it as their preferred HIV prevention strategy.^24,26–28,30,37,39,41^ Studies pointed to the challenges associated with the first pill-intake before a sexual encounter, which was dependent on planned rather than spontaneous sex,^27^ as well as forgetting to take any of the three doses required for full adherence.^24,27,28,39,41^ A young adult African American MSM in the US^24^ said that he forgot to take the pills “*in the heat of the moment*”, while another said, “*I would rather take it every day. … Taking two pills before this … It’s like asking me to cut the grass in the middle of my meal or something like that. It’s just not going to happen”.*^24^ One participant from a study in Australia^37^ noted how invasive the on-demand regimen was:

*“For the next 24, 48 hours, it’s kind of there stuck in my mind: ‘You must take this pill! You must take this pill!’ And in a way, it kind of goes back to how condoms used to be intrusive. It’s sort of re-established a bit of that idea of HIV prevention being intrusive instead of very much a passive thing that is going on in the background.”*

#### Domain 2 – compatibility with sexual lives

Compatibility with people’s sexual lives was noted in 23 studies, relating to diaphragms/caps,^17,19,21^ ED-PrEP,^24–31,33,35–41^ gels/films^43^ and ODCPs.^47–49^

##### Pregnancy prevention and intermittent planned or spontaneous sex

Diaphragms/caps^19,21^ and ODCPs^47–49^ were of particular appeal for women whose sex lives featured periods of intermittent or infrequent sex. Across studies and products, women did not see the need for a daily prevention product (which entails costs, daily action, medication exposure and potential side effects) when risk was intermittent or infrequent. In Niger,^21^ Caya diaphragm users explained that infrequent sex resulted from prolonged periods of geographic separation from partners, so they did not feel the need for daily protection for pregnancy prevention when they would not be having sex. For similar reasons, Haitian-American women in the US^19^ dismissed the need for routine injections and pills due to separation from partners due to insecure immigration status, family obligations in Haiti, or migration for employment.

Participants (women who have infrequent sex) in two studies exploring the acceptability of a pericoital LNG 1.5 mg pill in Ghana^48^ and Kenya^49^ expressed high satisfaction with the product. In Ghana, among the 873 women aged 18–49 years, “satisfaction” (97%) and “desire to use the method again in the future if available” (96%) were near universal.^48^ In these two studies, ODCPs were versatile enough to enable pre- and/or post-coital pregnancy prevention and cope with planned or spontaneous sex. In a study in Ghana and Zambia,^47^ married and unmarried women who reported infrequent or spontaneous sex were routinely using ODCPs as their primary contraceptive, preferring this to a daily method. One unmarried woman in Zambia said,

*“Sex just happens, its unpredictable, you could not predict it. So, it is better it is just home or in your handbag wherever you go, and that is not a problem. And if you have the finance supporting you, then you can buy as many as possible.”^47^*

##### HIV prevention and intermittent planned or spontaneous sex

ED-PrEP (typically indicated for men who have sex with men and transgender women not taking gender-affirming hormones, comprising two pills taken between 2 and 24 hours preceding sexual intercourse, followed by one pill 24 hours and another pill 48 hours after the first drug intake) appealed to some people who had intermittent or infrequent planned sex.^25,26,30,31,33,36,37,40,41^ In exploratory perception and preference studies in South Africa, Uganda and Zimbabwe,^30,33,36^ young heterosexual people (male and female) preferred ED-PrEP to daily PrEP (a single daily tablet taken at the same time each day). They felt they were not exposed to HIV frequently enough to warrant taking PrEP every day, not engaging in regular sexual activity, not living with their partner, faithful to their partners or tended to plan sexual activity in advance.^30,33,36^

Similar findings were seen in some studies with MSM where preference was bound up in associations between infrequent sex, the ability to plan for sex, and assessments of risk of HIV acquisition. In a Brazilian study with young MSM, *travestis* (people who were assigned male at birth and develop a feminine gender identity) and transgender women aged 15–19 years,^41^ one participant said, “*I think I would adhere to it. […] Even more so in the current situation. Because I’m not having much sex*”, while another said, “*Oh, I think it’s much cooler […] It’s very complicated for me to take medication every day. I just don’t like it*.” A man in a study from Canada^40^ explained that his doctor said he should transition to ED-PrEP from daily PrEP due to an issue with a blood test which showed changes with his white blood cell count. Once his tests returned to normal he said, “*It’s not like I’m sleeping around a lot, so I’ll just use [PrEP] on demand”.* Use of ED-PrEP due to predictability of weekend sex was also reported by MSM in studies in Belgium,^25^ the Netherlands^26^ and France.^27^

A preference for use of ED-PrEP also occurred at times of change in people’s sexual lives. In an Australian study,^37^ one participant switched to ED-PrEP after deciding he did not need daily PrEP because he lived in a rural area and only had sex with other partners during weekend visits to the city. In a study with gay, bisexual and queer men in Canada,^40^ one man explained that he shifted between daily PrEP and ED-PrEP dependent on his anticipated HIV risk exposure and sexual behaviours. A study with gay and bisexual men in Australia^31^ noted how a shift from daily PrEP to ED-PrEP occurred at particular life moments, including interstate moves, a return from living overseas or during COVID-19 social restrictions, where they were having less sex. One man said,

*“Everyone was in lockdown and things weren’t happening the way they used to. And I figured, ‘Well, why am I taking this every day? Because it’s highly unlikely that I’ll be needing this today or tomorrow, or the next day.’ It just didn’t make any sense continuing it daily.”^31^*

A transition to being in a longer-term relationship was another change. A man in the Netherlands explained that he was “*now in a monogamous relationship, so no risk for HIV”.*^26^ Conversely, in a study with MSM in Belgium, one man explained why ED-PrEP was not suited to his current stage of life:

*“I have a lot of routine in my life, but I have a hard, stressful and irregular job. [.] I think that daily [PrEP] is most safe, and that’s it. If you take the other [regimen] then you really have to have it planned to [take it] in advance, and that’s just really something I cannot do.”^25^*

In other studies, MSM^24,28,39^ and young people^29,30,36^ who reported unplanned, spontaneous sex were not interested in ED-PrEP. In a study with 140 MSM in the US,^28^ the most common barriers with ED-PrEP dosing included unplanned sexual encounters resulting in missing the double-dose pre-sex (43.6%) and trouble remembering doses post-sex (29.3%). In a study in Uganda, a young Ugandan woman said, “*I prefer daily PrEP because you never know when you are going to have sex. He may come abruptly when you have not yet taken on-demand PrEP*”.^30^ A young adult African American MSM in the US^24^ said, “*Because of the simple fact, our hormones, when people wanna have sex, they wanna have sex. I just feel like they never be thinking about a pill*”.^24^

##### Timing of product use

Characteristics associated with the timing of consumption of on-demand products were mentioned across a number of studies. Studies pointed to how timing of the first ED-PrEP dose is complicated due to the possible side effects and implications for sexual experience. In a South African study,^24^ one man said, “*If you take a pill before you have sex you don’t know how that’s gonna make you feel, you know, you might … you might be tired or something or might not be able to perform like you normally do”*.

In a study with Australian PrEP users,^37^ a man said he experienced stomach cramps within half an hour of taking the first dose of ED-PrEP, while another preferred to start his dosing cycle a day before he anticipated having sex, as he knew that feelings of nausea subsided a day after his first two pills.

Regarding ODCPs, a study in Ghana^48^ indicated high satisfaction and future use intentions among female participants due to the ability to take pericoital LNG 1.5 mg before sex. Haitian-American women participating in a FemCap study in the US^19^ liked the versatility of the cervical cap – both as an on-demand method, but also for longer periods of days if preferred. One woman said, “*some people sleep with it. After you have sex, the earliest you can take it out is 6 hours. But, if you want to go to sleep, shower, do regular activities for up to 2 days”.*^19^

In the Niger Caya study,^21^ women said that the 6-hour postcoital wait before diaphragm removal was a drawback of the method. In South Africa and Zimbabwe^43^ young women desired greater flexibility around timing of use of vaginal gels and films, which was not possible with coital dosing. Less than half (45%) of participants found the timing of the gel very acceptable, in part due to spontaneity of sex, but also preferring that products fully dissolve or disappear to be unnoticeable during sex.^43^ Some women wanted an option that gave greater potential for use that was not coitally-dependent, preferring that a film or gel could be used either up to one day or one week before sex.^43^

#### Domain 3 – side effects

Side effects of products were noted in 20 studies, relating to diaphragms,^17–19,21,22^ ED-PrEP,^25,26,28,31–33,35,37,39,40^ vaginal gels/films^42,44^ and ODCPs.^46,48,49^

##### Avoiding side effects

Preferred use of on-demand products to avoid side effects associated with other products were well documented in relation to ED-PREP^25,26,28,31–33,35,37,39^ and diaphragm/cap,^17,18,21,22^ ED-PrEP was perceived as being more acceptable than daily PrEP because products were used less regularly, leading to fewer perceived side effects, minimising the number of tablets consumed^25,26,32,35,37,39^ and limiting potential harm to the body due to pill toxicity.^25,26,31^ A man from an Australian study^37^ explained why he transitioned to ED-PREP from daily PREP:

*“If I start taking [daily PrEP], I feel sluggish. My stomach feels a bit crap. And if I take it before I sleep, I get such vivid dreams, like incredibly vivid dreams that I hate. I don’t know if that’s caused by PrEP but it feels to me like it’s caused by the PrEP ‘cause it only seems to happen if I take a pill right before bed. I guess as well that’s part of the reason why I’m not taking it daily, because I kind of feel I don’t really want to take it unless I have to, because it mostly just leaves my digestive system feeling ravaged.”*

A young man in a Zimbabwean study^33^ said,

*“We hear a lot of people in the community complaining that ever since they started taking pills, they have experienced side effects. That is one of the reasons why I prefer using them [PrEP] only on that day, not to take them daily. Using it [PrEP] for that day only will limit the amount of tablets in my body.”*

A reduction of side effects was also achieved through use of non-hormonal on-demand products. Women in studies in Uganda and Zimbabwe said they would use new vaginal gel products provided there were less frequent side effects and no menstrual changes.^44^ Women talked positively about the offer of an on-demand diaphragm/cap for pregnancy prevention compared with prior experiences they had had with hormonal contraceptives.^17,18,21,22^ In a Caya study in Benin,^22^, among 225 women who participated in the survey, the most popular reasons for using Caya were the absence of side effects (81%) and the non-hormonal nature of Caya (76%). In a study in Niger,^21^ many adopters of the Caya diaphragm recounted unsatisfactory experiences with other modern methods including oral contraceptive pills, injectables, and implants. One woman said, “*I saw that it's easier than pills. Pills give me a lot of problems”*, while another said, “*[With] the pill that I often take, in a single month I can see my period twice. That's why it's not easy and I changed [methods]”.*^21^ A woman in rural Uganda, talking about the SILCS diaphragm, said, “*This one will be good, if it has no side effects … Is there a woman who does not want family planning? Except it makes them sick”.*^17^ A Haitian-American women in a FemCap study said, “*I don’t want hormones, to prevent getting any more facial hair. I don’t want it. I am a woman”.*^18^

##### Tolerating mild side effects

Despite the perception that less regular dosing may diminish side effects relative to daily indications, research noted that on-demand products do not always eliminate side effects. This highlights trade-offs users often make in finding a product that works best for their lives and circumstances. In a study in the US,^42^ of the 1330 women who used a vaginal gel at least once, fewer than 2% discontinued due to adverse effects; worst symptoms were reported largely to be mild (23.9%) or moderate (18.7%) in severity. In an ODCP study in Ghana,^48^ vaginal bleeding (32%), headache (18%), cramps (14%) and nausea (9%) were the most common side effects. In this study, the majority of ODCP side effects were reported as mild or uncomfortable but tolerable (5% of all side effects were reported as intolerable), and satisfaction levels remained high.^48^

ED-PrEP studies^26,37^ revealed how MSM navigated side effects, and how on-demand dosing helped minimise the disruptions these entailed relative to daily oral PrEP. In an Australian study,^37^ one participant took ED-PrEP tablets with food to minimise nausea. But he explained that getting the timing of PrEP dosing “right” emerged not only as a matter of achieving effectiveness (i.e. at least 2 hours before sex), but also in achieving the “right” timing alongside other domestic routines and achieving other daily priorities (i.e. sleeping, eating, avoiding nausea or discreetly taking pills).

#### Domain 4 – accessibility of products

Accessibility of products was noted in 17 studies, relating to diaphragms,^17–21^ ED-PrEP,^23,26,28,29,31,34,37–39^ and ODCPs.^47–49^

##### Delivery channel preferences

Preferences for different providers and delivery channels were mentioned across product types. Research participants in some studies liked to access products from a variety of mainstream health services, including community health workers,^21^ facility-based providers or clinics,^18–21,28,29,34,47^ family planning clinics,^20^ HIV counsellors^29^ and hospitals.^39^ Participants in other studies liked more community-oriented services, including community-based organisations,^23,39^ PrEP navigators (i.e. community-experts with lived experience),^28^ pharmacists^29,47–49^ or drug stores.^47^ One study in Kenya provided evidence of feasibility via online providers.^49^

Preferences around preferred delivery channels were nuanced. In Niger, community health workers were valued by women using the Caya diaphragm because they enabled open conversations and opportunities to ask “trivial” or “intimate” questions.^21^ In Cambodia, a study with HIV key populations revealed that most participants liked community-based access to PrEP due to challenges associated with hospital-based PrEP which included a lack of explanation about PrEP intake, fear of discrimination, and feeling embarrassed in front of a doctor.^39^ However, the same study revealed that upper-class hidden MSM liked private hospital-based PrEP services because they were faster and more confidential.^39^

Research indicated evidence of acceptability and feasibility of pharmacy-led provision of ODCPs.^47–49^ In Ghana,^48^ over 99% of active participants stated they were satisfied or very satisfied with their pharmacy experience, while in Kenya,^49^ 83% were satisfied/very satisfied with an e-commerce service and delivery to participants’ choice of location, citing convenience, ease of use, and supportive/friendly staff. Unmarried women in a Zambian study^47^ highlighted the challenges associated with accessing on-demand contraceptive products in contexts of stigma around premarital pregnancy, with a strong preference for pharmacies or drug stores. As unmarried women aged 25–34 years from Zambia^47^ in a focus group explained,

*“In a chemist they will treat you well because they know that you will give them money, they will even explain to you. But at a government clinic, they can be hostile, they can even shout at you that you didn’t know, you can even get scared of going there.”*

##### Cost of products

Costs were reported as influencing choices about whether to use daily or on-demand products.^17,19,21,23,26,28,29,31,34,37,47,48^ Because of their as-needed nature, on-demand SRH products need to be easily accessible to users, which typically entails availability via the private sector (i.e. pharmacies, drug shops).

Diaphragms/caps were considered economical compared to other contraceptive options as they are durable and can be used for several years.^17,19,21^ For other products, the on-demand feature was seen as actually reducing costs to users by reducing the amount of product needed. For ED-PreP, for example, while the cost of the products might be the same as for daily PrEP, with on-demand options less product is required so the cost to clients may be perceived as less. ED-PrEP was perceived as cheaper than daily PrEP by participants in studies in the Netherlands,^26^ South Africa,^29^ Ukraine,^23^ Malaysia,^34^ Vietnam^38^ and Australia.^31,37^

There is some evidence that at least some users are willing to pay out of pocket for on-demand SRH products. In Ghana, 85% of the active participants said that they would be willing to pay for a pericoital ODCP if it was available on the market.^48^ “Willingness to pay” data was only mentioned in a Vietnamese ED-PrEP study in Vietnam (76.4% willing to pay if PrEP were less than US$15 per month),^38^ and a Ugandan SILCS study (women willing to pay from 0.12–6.00 USD, but prefer free).^17^

##### Pack size and multi-buy options

Pack size and multi-buy options were highlighted in ODCP studies.^47–49^ In ODCP trials, women were able to access up to six tablets, which was the maximum permitted dose in any 30-day period. In a study with unmarried people in Ghana and Zambia,^47^ multi-pack purchases were deemed highly acceptable because purchasing ODCPs this way would allow them to be always prepared. As one young man said, “*Unplanned sex is why I was saying you need to buy in bulk, because you don’t know when fire will come. It is always better to prevent than to cure”.*^47^

#### Domain 5 – compatibility with daily life

Compatibility of on-demand products with people’s lives within particular local social, cultural and religious contexts was noted in 13 studies, relating to diaphragms/caps,^17,19,21^ ED-PrEP,^23,30,33–36,39^ gels/films^44^ and ODCPs.^46,47^

##### Preferences within stigmatised contexts

Contexts of stigma and discrimination influenced choice of ED-PrEP^23,30,33–36,39^ and ODCPs. ^46,47^ Risks of stigma were experienced by young women considering routine post-coital ODCP use in Ghana and Zambia, associated with social values that prohibit and stigmatise sex before marriage.^47^ With regard to HIV prevention, ED-PrEP was preferred by particular people who experience, or reported risk of experiencing, stigma related to HIV or same-sex sexuality in diverse contexts. A discrete choice experiment in Ukraine indicated high preference for ED-PrEP among a particular group of MSM who had high levels of internalised homophobia.^23^ A discrete choice study in Malaysia identified strong preference for ED-PrEP among a particular group who were more likely to be Malay and less likely to come out to their families.^34^

Young people in southern African settings^30,33,36^ and HIV key populations in Cambodia^39^ preferred ED-PrEP over daily PrEP to avoid HIV stigma associated with being seen consuming medication that others might assume was antiretroviral therapy pills. A young person in a mixed gender focus group in South Africa said, “*sometimes I am out partying, and I have to take the pills. Some people, they jump into conclusions there and there that [name] is positive”.*^33^

##### Influence of socio-cultural values

Four studies noted the need to understand how preferences for SRH products aligned within socio-cultural contexts.^17,19,21,44^ In the Niger Caya study, men said that leaving Caya inserted for six hours after sex conflicts with the Islamic religious practice of cleansing one’s body before prayer.^21^ Two studies^17,44^ situated vaginal gels and lubricants within cultural contexts associated with sex. In a Ugandan study on the SILCS diaphragm,^17^ women agreed that some type of lubricant was necessary to help with diaphragm insertion, but noted this aligns with cultural practices where women use herbs to enhance vaginal lubrication in some settings (i.e. central and western Uganda) but conflicts with use of herbs to dry the vagina in other places (i.e. northern Uganda). Similar findings about the use of lubricants not being equally acceptable across different settings were found in a discrete choice experiment about gels in Zimbabwe and Uganda.^44^

#### Domain 6 – partner dynamics with product use

Partner influence on use of on-demand products was noted in 10 studies, relating to diaphragms,^17–22^ ED-PrEP,^37^ and gels/films.^43–45^

##### Discreet, independent or shared decisions

Participants in some studies viewed on-demand self-care products as discreet or covert, enabling some women to have greater control over their own lives in contexts where gender norms enabled men to control decisions constraining their sexual and reproductive agency. Covert use of the FemCap cervical barrier was noted by Haitian-American women.^18,19^ Thirteen of the 20 women in the study did not discuss the device with their partner, and five of the nine women who used it during sex did so covertly, without prior partner negotiation.^18^ When one woman told her partner about the device, he reiterated that he wanted to have children. She told him she would stop using the device but revealed in a focus group that she intended to keep using it due to her ambivalence about having children.^19^ One married woman said, “*my husband still can’t tell when I wear [Femcap]. And I am protecting myself. I am not having the kids that he wants right now. And basically that’s it”.*^18^

In contrast, for some study participants, decisions around choice and use of on-demand products were shared. In a SILCS diaphragm study in South Africa, most women used the study products at their partner’s home and talked with their partner about the products at each use.^20^ In the Niger Caya study, most adopters (18 of 25) reported that their male partner was involved in using, seeking and paying for the device; in one case, a man said he helped with insertion and removal.^21^ One young woman described her partner’s response to her interest in Caya: “*He said it is good. You know, nowadays no one likes closely spaced pregnancies”.*^21^ In Benin,^22^ some men also assisted women with Caya insertion.

With regard to use of ED-PrEP, for one man and his husband in a study in Australia^37^ shared decisions and this afforded them excitement:

*“It’s like ‘hey, we’ve decided we are going to have this guy over and we are going to do this, yay, let’s take PrEP’. It’s like an advent calendar [laughter]—that’s such an inappropriate analogy—but it’s like ‘this is day 1, this is day 2, let’s fuck’ and a couple of days after, you keep going [with PrEP]. So, to me it’s actually part of the build up of ‘we get to do that this weekend’.”*

##### Sexual pleasure

Independent and shared decisions about the use of vaginal products were also reported as influencing people’s pleasurable sexual experiences.^17,19,21,22,43–45^ In a trial of a non-hormonal, on-demand, lubricating contraceptive gel in the US,^45^ results indicated high and increasing levels of sexual satisfaction over the study duration, alongside significantly decreasing experiences of sexual problems including vaginal dryness, lack of sexual interest and/or desire, vaginal tightness, pain, anxiety, inability to orgasm, and vaginal bleeding or irritation. In a FemCAP study, some female participants said that use of the cervical cap aligned with their male partner’s preferences. One woman said her partner was supportive because he “*was tired of pulling out”*, and another had a partner who did not want to use condoms any more.^19^ Among Caya users interviewed in Niger, several appreciated the lubricating effect of Caya gel, with one woman explaining, “*it doesn’t annoy us. He [my husband] loves it”.*^21^ Similar findings were seen in the Benin Caya study too.^22^

However, other products were viewed differently between sexual partners. In a study about vaginal gels in South Africa and Zimbabwe,^43^ young women described the gel being easily felt by their partner due to the increased wetness it caused. For some, this was a benefit, increasing pleasure during sex, while for others, this caused concerns. One young Zimbabwean woman said, “*For me gel was easy on application, but it was not as good on sex because my husband would say it feels like you already had sex, he did not like that slipperiness”.*^43^

## Discussion

The objective of this systematic scoping review was to undertake a comprehensive review of available published research to explore the acceptability of SRH on-demand self-care from the perspectives of users across diverse regions of the world. Studies from 20 countries across four WHO regions point to a strong acceptability of on-demand self-care SRH products.

Our work has revealed six emergent domains that can be used to enhance understanding of the role that on-demand SRH products play in users’ lives: ease of use; compatibility with sex lives; side effects; accessibility of products; compatibility with daily life; and partner dynamics with product use. While studies were not longitudinal in nature, our analyses also highlight that interest in and experiences with such products vary for people in different phases of their life course, according to changes in their circumstances, relationships and sexual practices.

These domains were identified through inductive analyses of primary data presented in published papers. This person-centred approach illustrates the potential for SRH-oriented research and on-demand product design that captures issues perceived as most important by users and potential users themselves, as opposed to those predetermined as priorities by researchers, funders or policy makers.

### How does use of on-demand SRH products enable self-care?

In answering our second research question, we reveal four cross-cutting themes where on-demand SRH products enable self-care: enhanced choice and agency; people-centred products; people-centred SRH service delivery; and more pleasurable, safer approaches to SRH.

#### Choice and agency

Analyses illustrate that wider availability of on-demand self-care products could enable people with more choice and agency in decision making around their SRH needs and wellbeing.^2,3,5,7^ Use of on-demand products improved flexibility, convenience, opportunity and choice in the way participants in diverse studies navigated SRH risks associated with HIV or pregnancy. These types of products allow people to take control of their health and use products only when or where they are needed. Importantly, these products support autonomy around how and when users disclose their use. These decisions and actions can be something done together with a partner, if that’s what people want, or on their own, if they choose or need to use products to gain more control over their sexual and reproductive lives. Products designed for private use mean that sexual relationships and conversations about contraception can happen at a time and in a manner that feels right for the user.

Focusing on choice brings attention to the well-considered trade-offs that people make to meet their needs within the realities of their lives and circumstances. On-demand products give people choice, which, alongside other SRH products, allows them to find products that work best for them at that time in their life. The data on side effects – relating to diaphragms,^17–19,21,22^ ED-PrEP,^25,26,28,31–33,35,37,39,40^ vaginal gels/films,^42,44^ and ODCPs^46,48,49^ – highlight this clearly: study participants were weighing and making trade-offs across a wide number of perceived attributes (among which effectiveness was just one attribute of importance) when making product choices. Each individual’s assessment of these attributes is unique, reflecting personal priorities and contexts.

#### People-centred products

On-demand SRH products can support people-centred SRH care.^2,3,5,7^ The addition of this product category to SRH services acknowledges the necessity to be attentive to diverse needs, desires and emotions around SRH, the relationality of care seeking and self-care, as well as experiences and priorities across contexts, identities and different stages of the life course. For example, papers reported that, for some, diaphragms,^17–21^ ED-PrEP,^37^ and gels/films^43–45^ enabled discrete prevention of HIV acquisition or unintended pregnancy. But the need for product discretion differed. For some the aim was to avoid family or community stigma related to premarital pregnancy^46,47^ or HIV infection;^23,30,33–36,39^ on-demand products enabled agency to help people navigate socio-cultural contexts of constraint, stigma and discrimination. For others, the aim was to be discrete within intimate sexual relationships. For example, Haitian-American women^18,19^ used FemCap without partner knowledge to navigate differing desires to have children. In this way, assessments of product attributes related to whether a product could be used discreetly differed between people, and product characteristics (such as possibility for covert/discrete use) are not stable but subjective, differing between and even within individuals depending on their circumstances and priorities. Either way, the inclusion of on-demand products for SRH enabled people to practice more agency within complex social contexts, choosing products that worked for them, at that time in their lives, and enabled them to navigate social pressures within close relationships and specific cultural contexts.

Another example of the people-centredness of on-demand products was the transition to and from these types of products, depending on different events in people’s life course. Use of ED-PrEP was perceived as beneficial for some people who were having less regular, more spontaneous sex, as it was for others who were having less regular, more planned sex. A transition from daily PrEP to ED-PrEP coincided with people moving house, becoming involved in long-term relationships, as well as navigating the reduction of social and sexual networks during COVID-19 lockdowns. A life course perspective reemphasises the need for choice of products (including on-demand products alongside other offerings) that are as aligned with people’s sexual lives as they are compatible with their daily lives, and acknowledges that preferences will change over time.

#### People-centred SRH service delivery

The availability of and access to on-demand products enables a shift from SRH care that is led by health services, to a more people-centred, decentralised and versatile form of service interaction that allows people to choose the extent to which they want to engage with formal health services.^2,3,5,7^ For instance, such a shift has been seen in the latest WHO guidelines on post-exposure prophylaxis (PEP), which recommends delivery of PEP in community settings and via task sharing involving non-specialist health workers.^50^ While on-demand products enable forms of self-care that do not require the support of a health worker, user perspectives pointed to diverse preferences for engagement with health systems. Some participants liked to access on-demand products from varying channels of formal health services,^18–21,28,29,34,39,47^ while others liked care from pharmacies, online, e-commerce or community-led initiatives.^23,28,29,39,47–49^ This aligns with calls for more people-centred care to ensure SRH services are provided at the right time, in the right place and in the right way, aligned with the needs and preferences of communities, and in doing so to support efforts towards universal SRH coverage and equity.^6^

System-level planning that enables easier, discreet or safer access to on-demand products from a range of public and private providers could also be factored into decisions about how best to bring new products to market in diverse social settings around the world. On-demand products present health systems challenges, including access, costs (to both health systems and end-users), patient and provider training and capacity-building approaches, and the complexity of administration and use (which have implications for product safety and efficacy as a self-care option, as well as provider training). Moving care closer to end-users also means consideration of private sector service delivery channels and may require both demand- and supply-side actions to ensure that high-quality products are accessible, affordable and equitable. Regardless of the channels these products are introduced in, from our analyses, it remains clear that there remains a role for healthcare providers, pharmacists and other health actors as gateways to self-care products, provided that they do so without assumptions or stigmatisation about which products are suitable for which groups of users. They remain important as sources of information, training on how to use products, for answering questions, dealing with side effects, and access to the products themselves.^18–21,31,48,49^ Positive experiences with on-demand self-care and product provision may also form a trusting bridge to other longer-acting/more effective methods when and if people want these.

#### Putting the pleasure back into SRH

Meaningful efforts to prevent unwanted or unintended outcomes associated with sexual intercourse occur at the intersection of sexual health, sexual rights and sexual pleasure.^51^ What seems very clear from this analysis is that, for many people, on-demand SRH self-care products put the pleasure back into SRH, while supporting people’s SRH rights. In 2006, the WHO published working definitions of sexual health as “a state of physical, emotional, mental and social wellbeing in relation to sexuality”, and sexual rights as including the right of all persons to “pursue a satisfying, safe and pleasurable sexual life”.^52^ A WHO update in 2022^53^ noted the importance of promoting intimacy, pleasure, consent and wellbeing at all stages of the life course, through adolescence and into older age.

Our findings indicated that the availability of, and opportunity to use, on-demand products can enhance people’s sexual health and wellbeing through safer, more pleasurable sex. This was illustrated through: improved compatibility of existing SRH products with people’s sex lives; more acceptable experiences with, or the removal of, side effects; product characteristics that helped women and their sexual partners, independently and/or jointly, enjoy more pleasurable sex and plan for and enjoy sex free of unwanted social or health consequences; navigate complex partner dynamics and negotiations, either discreetly or by enabling new opportunities for communication; and adapt to ongoing challenges and changes of daily life that arise in specific settings. While SRH self-care interventions on their own may not address the full scope of barriers to sexual health, user experiences and preferences suggest these options have the potential to enhance pleasure and extend sexual well-being for couples and individuals.

### Study limitations and strengths

There are some limitations to this review. We report on 33 papers that documented user perspectives on four on-demand SRH self-care products, which is a limited resource base. The availability of published literature varied for each product; there were greater numbers of published papers for products that have launched and been in market (i.e. ED-PrEP) compared with newer products (i.e. ODCPs). We have only reported on studies published in peer-reviewed journals and excluded grey literature including government and community reports. The aim of this review was not to assess the quality of the research involved, but to identify and summarise key themes.

There are several key strengths to this paper. For example, we used multiple databases to identify all available papers that might help us to answer the research questions; there was dual review of all papers through the inclusion and exclusion process, as well as detailed review of the final selected papers; we brought a rigorous, novel inductive thematic analysis process to analyse included papers, which enabled us to answer the research questions with original insights. Through this process, we have identified themes that are common to on-demand products across diverse studies in a wide range of settings, which has shed light on user perspectives concerning on-demand SRH products.

### Implications for future research

The rights of individuals and couples have been central to SRH practice since the 1990s, with issues of autonomy, agency and universal access to modern contraceptives and related services firmly established in the United Nations Sustainable Development Goals.^8,54^ This category of SRH on-demand self-care products could enable people, with support, to take more control over their sexual and reproductive health – through access to products that are better aligned with their personal needs and preferences, the dynamics of their sexual relationships, or constraints arising from the social contexts in which they live. That said, the impact of this new category of SRH products is constrained by a variety of influences, such as the cost of products, gender and social norms that influence autonomy in decision making, laws and policies that influence the introduction and availability of new SRH product types, and ability of health systems to integrate new products into everyday service delivery. The result is further risk of inequity in access to products across different populations, with those most in need of product diversification least likely to benefit from the opportunities that they present.

With these issues in mind, a range of avenues for future research can advance this work exploring the possibilities of SRH on-demand self-care products. These include a focus on: (1) building the evidence base around the importance of user research in all stages of SRH product design; (2) user-centred research to enhance access to and use of on-demand self-care products, including understandings of affordability (i.e. self-care cannot equal self-pay) associated with private sector models, and preference for over-the-counter, prescription or community-based supply models to de-medicalise time-dependent, on-demand products; (3) modelling and resource allocation research which balances costs and health/social impacts associated with introducing on-demand products to optimise national-level public sector investment; (4) implementation research to finetune effective approaches to delivering existing, newer and in-development on-demand products; (5) initiating a life stage analysis of on-demand SRH care and associated packages of support, establishing how integrated health systems can support users (e.g. through referral pathways, with guidance and advice) as they transition in and out of use of on-demand and daily products; and (6) research to tackle the geographical gaps in diverse social, cultural and political settings in Southeast Asia and Eastern Mediterranean Regions.

## Conclusions

We believe there is value in conceptualising an “on-demand SRH self-care” category, with regard to both types of product and approaches to SRH service delivery. For instance, despite the differences in products used to prevent pregnancy or HIV infection, which are often siloed in product development as well as policy and programmes, a unifying category such as this has utility. Programmes for HIV, sexually transmitted infections, maternal and child health, antenatal care and family planning tend to be funded and administered separately. Yet this is not how people and families experience these areas of health and wellbeing. More broadly, this categorisation helps draw attention to the benefits of people-centred approaches to SRH service delivery and care, which are integral to efforts to achieve universal health coverage. Addressing on-demand SRH self-care permits a view across the SRH space, and opportunities to address commonalities and help empower users to have satisfying sex lives, plan the families they want, stay healthy, and have the partnerships they desire.

Pragmatically, the six emergent domains that we have identified can be used in different ways to support people with more choice in SRH services and care through a more user-centred lens on new product introduction and scale-up, as well as adaptation of existing products that are available in international markets. In particular, these domains could be used strategically to position existing and new on-demand products into the market; engage and communicate with end-users in ways that enhance their use of safe, effective products most aligned to their needs and preferences across the life course; advocate for changes in policy and regulatory environments that are more supportive of on-demand SRH products; and prioritise future investment in the on-demand product category.

## Supplementary Material

Supplemental material. Preferred Reporting Items for Systematic reviews and Meta-Analyses extension for Scoping Reviews (PRISMA-ScR) Checklist

## Data Availability

No primary data was used.
